# Effect of nicotine on biofilm formation of *Streptococcus mutans* isolates from smoking and non-smoking subjects

**DOI:** 10.1080/20002297.2019.1662275

**Published:** 2019-09-13

**Authors:** Nasreen F. El-Ezmerli, Richard L. Gregory

**Affiliations:** a Department of Operative and Preventive Dentistry, School of Dentistry, Indiana University, Indianapolis, IN, USA; b Department of Biomedical and Applied Sciences, School of Dentistry, Indiana University, Indianapolis, IN, USA

**Keywords:** Biofilm, tooth decay, *Streptococcus mutans*, nicotine

## Abstract

**Objectives**: To investigate effects of nicotine on biofilm formation of *Streptococcus mutans* isolates from oral washes of smoker and non-smoker human subjects.

**Materials and methods**: This study was conducted using 60 *S. mutans* isolates with three *S. mutans* isolates collected from oral washes of ten smoking subjects and ten from non-smoking subjects. Biofilm was formed by culturing each *S. mutans* strain (10 μl) in 190 μl of TSB supplemented with 1% sucrose (TSBS) containing 0, 0.25, 0.5, 1.0, 2.0, 4.0, 8.0, 16.0, and 32.0 mg/ml of nicotine for 24 h in 5% CO_2_ at 37°C in 96 well microtiter plates. The absorbance values of biofilm were measured at 490 nm in a microplate spectrophotometer.

**Results**: There was a significant effect (p-value < 0.05) of nicotine concentrations and smoking on the growth of biofilm, planktonic cells, and total absorbance, for all strains of *S. mutans*. Isolates from smokers had significantly more biofilm at 0–16 mg/ml of nicotine compared to those from non-smokers (p-value < 0.0001).

**Conclusion**: *S. mutans* smoker isolates are more affected by high nicotine concentrations than non-smoker isolates.

**Clinical Relevance**: The use of nicotine products increases the growth of *S. mutans* and may place tobacco users at risk for dental decay.

Tooth decay is a complex dieto-bacterial disease with an association of social, behavioral and biological factors [1]. This complex disease is considered an infectious disease, which develops over time involving a complex interaction of oral microflora, specifically *Streptococcus mutans*, dietary carbohydrates, and a susceptible tooth surface [2]. It has been well defined that *S. mutans* and tooth decay are closely related, especially that *S. mutans* can adapt very well in a high carbohydrate environment under acidic conditions. *S. mutans* has the ability to metabolize sugars forming organic acids that bathe tooth surfaces causing its progressive mineral loss. It thrives in specific oral conditions with unique characteristics [3]. Adherence of *S. mutans* to hard tooth structures is considered one of the major characteristics that enable it to proliferate and microcolonize establishing a mature cariogenic biofilm. Numerous cariogenic factors of *S. mutans* are involved in its ability to adhere and aggregate to form cariogenic biofilms including initial sucrose-independent adherence in which antigen I/II is involved, and sucrose-dependent adherence based on the function of glucosyltransferases (Gtfs) and other glucan-binding proteins (Gbp) [3]. Oral microbial biofilm (dental plaque) formation involves four stages including salivary acquired pellicle formation, microorganism adherence, growth and maturation of the bacterial microcolonies, and lastly detachment to form a new biofilm [4]. In the first stage, if the tooth surface is clean, salivary molecules can adsorb to hydroxyapatite on enamel tooth surfaces by electrostatic interactions forming the acquired enamel pellicle. Initial microorganism adherence is the second stage that occurs when early colonizing bacteria attach to salivary acquired pellicle through a weak reversible attachment in the absence of sucrose utilizing specific receptors and ligands [4]. *S. mutans* has an important role in initial sucrose independent adherence involving a bacterial surface protein adhesin called antigen I/II that interacts specifically with a high molecular weight salivary agglutinin glycoprotein (SAG) found in the acquired enamel pellicle [5,6]. The third stage involves formation of an extracellular polysaccharide matrix and establishment of cariogenic biofilm attached to tooth surfaces which is contributed by an important cariogenic factor of *S. mutans* known as sucrose-dependent adherence involving Gtfs and Gbps [7,8]. *S. mutans*-associated Gtfs primarily produce both water soluble and insoluble glucans by metabolizing sucrose to glucose and fructose and subsequently polymerizing glucose to an extracellular adhesive insoluble glucan that binds bacterial cells together through Gbp and Gtf receptors and adhere the cells to the enamel tooth surface [8]. It was observed that deletion of the *Gtfs* genes remarkably decreases the cariogenic potential of *S. mutans* strains [9]. The synthesis of extracellular glucan enhances the adherence of *S. mutans* through a cell to cell interaction where streptococcal Gtfs bind to glucan that successively adheres cells to smooth tooth surfaces [10]. Additionally, *S. mutans* synthesizes Gbps that have a significant role in establishing a mature biofilm by adhering bacteria to the extracellular glucan. An *in vitro* study by Lynch et al. indicated that engineered *S. mutans* with deleted Gbps genes affected the adherence and aggregation of these organisms resulting in a decrease in the biofilm mass and change in its architecture [11].

Tobacco use is a behavioral risk factor that adversely affects oral health and is directly linked to common life threatening diseases such as cancer, and cardiovascular and respiratory diseases [12–14]. The oral cavity is the first place in the human body to get exposed to either chewing tobacco or tobacco smoke and its chemical components. Therefore, tobacco not only affects systemic organs but it also has a significant influence on periodontal and other oral tissues [15] as well as oral microorganisms. Nicotine is one of the major active ingredients of cigarette smoke [16]. This active chemical has a toxic effect on alveolar bone and clinical attachment loss [17]. The exact effects of nicotine associated with tooth decay has not been fully investigated. However, a study conducted on 824 male Mexican truck drivers found a remarkable association between tobacco use and dental caries experience. Drivers who smoked more than 10 cigarettes/day had twice as many carious lesions than non-smokers [18]. In an Italian military population it was determined that heavy smokers had double the number of decayed teeth than a general population [19]. Another study investigated the *in vitro* effect of cigarette smoke on the growth of *S. mutans* and *Streptococcus sanguinis*. They concluded that nicotine has a dose-dependent effect on the growth of *S. mutans*; since as the nicotine concentration in the cigarettes increased there was an increase in *S. mutans* growth [20]. In an *in vivo* study, it was reported that nicotine treated rats had a significant increase in *S. mutans* growth and developed more caries lesions than in nicotine untreated rats [21]. Recently, we determined that nicotine stimulates *S. mutans* planktonic cell Gtf and Gbp expression as a mechanism to increase planktonic cell attachment to biofilm matrix leading to an increased number of cells in the biofilm [22]. This may explain the development of more carious lesions in smokers. In another study from this laboratory, seven *S. mutans* strains were treated with different nicotine concentrations (ranging from 0–16 mg/ml) [23]. Biofilm formation, and metabolic activity of the strains were determined. Biofilm formation and metabolism of all seven *S. mutans* strains increased in a dose-dependent manner up to 16.0 mg/ml of nicotine. Planktonic cell growth exhibited the highest values between 2, 4 and 8 mg/ml of nicotine. Because of these significant effects of nicotine on *S. mutans*, it is possible that there may be a difference in the manner that *S. mutans* responds to nicotine in smokers. To date there is no information on the effect of nicotine on the biofilm formation of *S. mutans* isolates from smokers. Therefore, we proposed the use of an *in vitro* model to better understand the effects of nicotine on biofilm formation of *S. mutans* isolates from smokers and non- smoking subjects.

## Materials and methods

### Bacterial strains and media

Ten oral washes collected from smoking subjects and ten oral washes from non-smoking subjects were used in this study. Three *S. mutans* isolates were cultured from each oral wash. Therefore, a total of 30 presumptive *S. mutans* smoker isolates and 30 *S. mutans* non-smoker isolates were collected. The oral washes were collected as part of a large multicenter NIH-funded microbiome grant (HL098960) and were obtained under appropriate IRB approval (IRB number 1,401,371,742). Age, race, gender, smoking history and number of pack years history were obtained from each subject. The oral wash samples were stored at −80°C until used. Selective agar plates (MSSB; Mitis Salivarius Sucrose Bacitracin; Anaerobic Systems, Inc., Morgan Hill, CA) were used for culturing the oral wash samples in 5% CO_2_ at 37°C as an initial isolation step, and three different colonies representing *S. mutans* from each oral wash sample were selected and grown on different MSSB plates. The isolates were subcultured in tryptic soy broth (TSB, Acumedia, Baltimore, MA) for 24 h in 5% CO_2_ at 37°C. The isolates were stored in TSB with 20% glycerol at −80°C until used. Mannitol and raffinose carbohydrate fermentation assays were used to confirm the identity of the subcultured *S. mutans* isolates [24]. A total of 34 *S. mutans* isolates were confirmed (11 from smokers and 23 from non-smokers) from a total of 60 non-confirmed *S. mutans* isolates (30 from smokers and 30 from non-smokers). Nicotine from Sigma-Aldrich (St. Louis, MO) was used.

### Biofilm formation

Overnight cultures of each *S. mutans* strain (10 μl representing approximately 10^6^ bacteria) grown in TSB were incubated with 0, 0.25, 0.5, 1.0, 2.0, 4.0, 8.0, 16.0, and 32.0 mg/ml of nicotine in TSB containing 1% sucrose (TSBS; 190 μl) for 24 h at 37°C in 5% CO_2_ in sterile 96 well microtiter plates (Fisher Scientific, Newark, DE). The total absorbance of each sterile 96 well microtiter plate was measured at 595 nm in a microplate spectrophotometer (SpectraMax 190; Molecular Devices, SunnyVale, CA) to assess the total bacterial growth (planktonic + biofilm cells). One hundred and twenty μl of planktonic cells of each sterile 96 well microtiter platewas transferred to other microtiterplates and the planktonic cell absorbance was determined at 595 nm. The biofilm remaining in each sterile 96 well microtiter platewas washed twice with deionized water, fixed with 200 μl of 10% formaldehyde (Sigma) for 30 min at room temperature, and washed three times with dionized water. Two hundred μl of 0.05% crystal violet was used to stain biofilm cells for 30 min. The wells were washed three times and 200 μl of isopropanol (Fisher, Pittsburg, PA) added for 60 min to extract the crystal violet from the biofilm cells. The absorbance values were measured at 490 nm.

### Statistical methods

Each of the 34 confirmed *S. mutans* strains was tested three times in quadruplicate. Summary statistics (mean, standard deviation, standard error, range) of the absorbance values (total absorbance, planktonic and biofilm) were calculated for each of the strains. The effects of nicotine concentration, smoker vs. non-smoker *S. mutans* strain, and their interaction on biofilm formation were analyzed using ANOVA. The ANOVA included fixed effects for the two factors and their interaction and a random effect of absorbance values were examined. A transformation of the data (e.g. natural logarithm) was necessary to satisfy the ANOVA assumptions.

## Results

**Figure 1.**
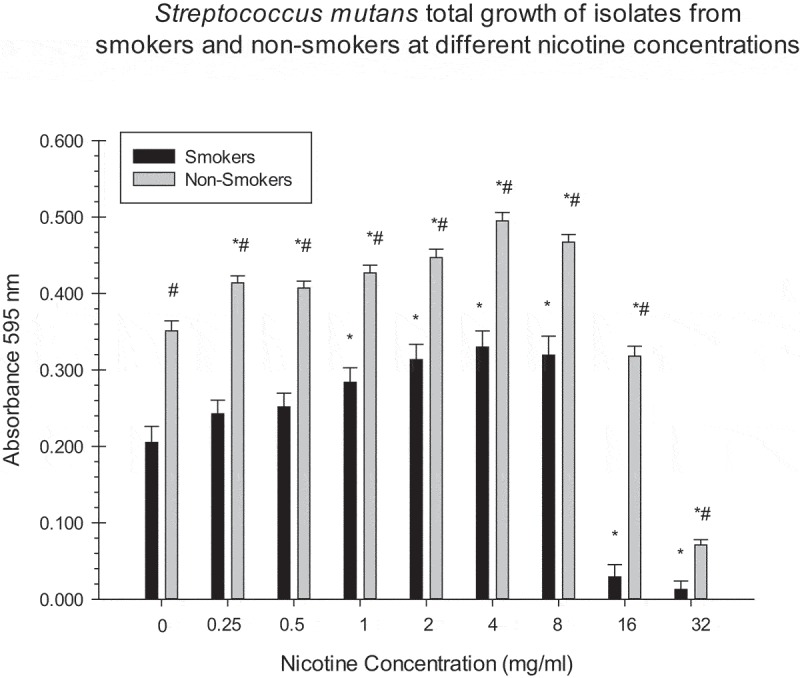
Asterisks indicate significant differences between total growth of *S. mutans* isolates (smokers/non-smokers) at different nicotine concentrations and the zero nicotine control. # indicate significant differences between total growth of *S. mutans* isolates of smokers/non-smokers at different nicotine concentrations.

**Figure 2.**
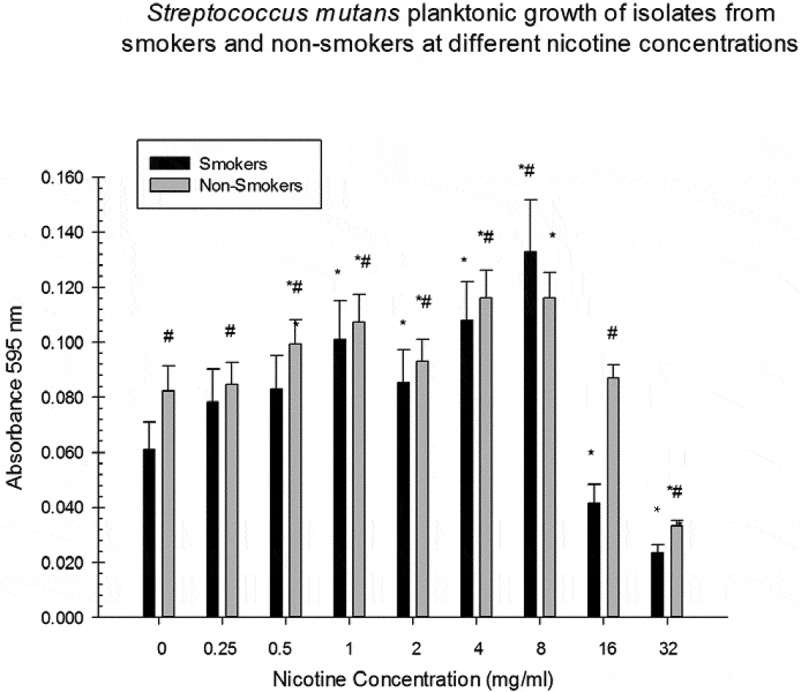
Asterisks indicate significant differences between *S. mutans* planktonic growth (smokers/non-smokers) at different nicotine concentrations and the zero nicotine control. # indicate significant differences between *S. mutans* planktonic growth of smokers/non-smokers at different nicotine concentrations.

**Figure 3.**
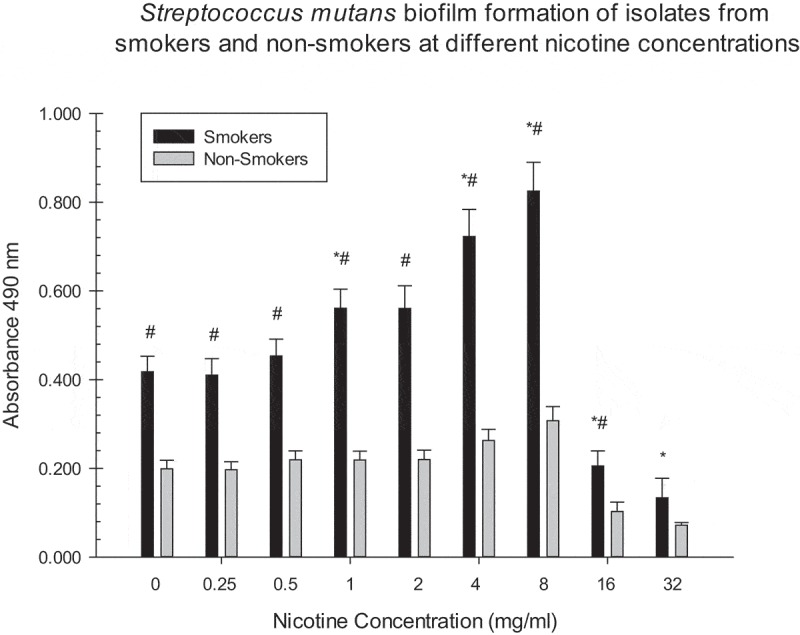
Asterisks indicate significant differences between *S. mutans* biofilm formation (smoker/non-smoker) at different nicotine concentrations and the zero nicotine control. # indicate significant differences between *S. mutans* biofilm formation of isolates from smokers and non-smokers at different nicotine concentrations.

Due to non-normality of the data, a rank transformation was used on the data prior to the analysis. A two-way ANOVA with a random effect for the multiple experiments was used for the analysis. There were significant effects of both nicotine concentrations and smoking on the growth of biofilm, planktonic cells, and total absorbance, for all strains of *S. mutans* (p < 0.0001; Figure 1–3). For biofilm, there was a significant interaction of nicotine concentrations and smoking for *S. mutans* smoker strains (Figure 3). For planktonic and total absorbance, there was a significant interaction of nicotine concentration and non-smoking *S. mutans* isolates (p < 0.0001; Figures 1 and 2, respectively). Biofilm formation of smoker isolates had dose-dependent effects up to 8.0 mg/ml. Isolates from smokers had significantly more biofilm at 0–16 mg/ml nicotine compared to those from non-smokers (p < 0.0001). Non-smoker isolates had significantly more total absorbance at all nicotine concentrations compared to smokers (p < 0.0001; Figure 1). There were significant differences of the planktonic cells between smoker and non–smoker isolates (Figure 2).

There were significant differences between biofilm formation of smoker isolates at 1, 4, and 8 mg/ml of nicotine and the zero nicotine concentration (Figure 3). Significant differences between biofilm formation of non-smoker isolates and the zero nicotine concentration were observed at the 4, 8, 16, and 32 mg/ml concentrations. There were significant differences between biofilm formation of smoker and non-smoker isolates at 0.25, 0.5, 1, 2, 4, 8, 16 and 32 mg/ml nicotine concentrations (Figure 1). For total absorbance and planktonic measurements there were significant differences between smoker and non-smoker isolates at all nicotine concentrations (Figures 1 and 2). There was no significant relationship between the number of pack years smoked (Tables 1 and 2) and biofilm formation of *S. mutans* isolates at all nicotine concentrations. However, this correlation was statistically significant (negative) for the 0.25 mg/ml nicotine concentration for one of the three experiments. Several other correlations indicated some relationship although they did not reach statistical significance.

**Table 1.** Average Demographic Factors of Smoking and Non-Smoking Human Subjects.

Demographic Factors	Smokers n = 10	Non-Smokers n = 10
Gender	F = 1 M = 9	F = 4 M = 6
Average Age	40.5 years old	42.0 years old
Race	White = 1 African American = 9	White = 5 African American = 5
Average: Pack Years	(8.5 cigarette per day/20) x 34.3 year smoking history = 14.5 pack years

**Table 2.** Individual Demographic Factors of Oral Washes from Smoking and Non-Smoking Human Subjects.

IUPUI- ID #	Race	Sex	Age	Smoker	Smoking History	Pack Years
891084OR01	W	M	52	No		
891080OR02	W	M	32	No		
891086OR01	AA	F	38	No		
891090OR01	AA	M	37	No		
891087OR01	AA	M	40	No		
891085OR01	W	M	22	Yes	½ PPD~4 years	3 years
891091OR01	W	M	52	No		
891088OR01	AA	F	42	No		
891089OR01	W	M	35	No		
891092OR01	W	F	56	No		
005017OR01	AA	M	54	Yes	1 PPD~36 years	36 years
005022OR01	AA	M	43	Yes	3 cig PD~42 years	6.3 years
005016OR01	AA	F	46	No		
005009OR01	AA	M	53	Yes	1 PPD~32 years	32 years
005010OR01	AA	F	48	Yes	8 cig PD~30 years	12 years
005011OR01	AA	M	51	Yes	1 PPD~37 years	37 years
005020OR01	AA	M	53	Yes	15 cig PD~34 years	25.5 years
005021OR01	AA	M	57	Yes	½ PPD~43 years	22.5 years
005024OR01	AA	M	58	Yes	1 PPD~41 years	41 years
005025OR01	AA	M	59	Yes	½ PPD~44 years	22 years

## Discussion

To determine the effect of smoking history and the addition of nicotine on the formation of *S. mutans* biofilm, planktonic cells, and total growth *in vitro, S. mutans* isolates from smokers and non-smokers were compared in this study. To date, this is the first study that compares the effect of nicotine on both smoker and non-smoker isolates. In this study, nicotine enhanced biofilm growth in both *S. mutans* smoker and non-smoker isolates. Biofilm formation increased in a dose-dependent manner up to 8.0 mg/ml nicotine in both smoking and non-smoking oral strains. Furthermore, smoker isolates, when incubated with most of the nicotine concentrations, produced significantly more biofilm compared to the non-smoker isolates. However, the total growth of the non-smoking isolates was significantly more than smoker isolates at several nicotine concentrations. This is consistent with a previous *in vitro* study from this laboratory reporting that biofilm formation and metabolism of *S. mutans* increased in a dose-dependent manner up to 16.0 mg/ml of nicotine [23]. Planktonic cell growth was highest between 2, 4 and 8 mg/ml nicotine. The majority of isolates had MIC values of 16.0 mg/ml nicotine, MBC of 32.0 mg/ml nicotine, and MBIC of 16.0 mg/ml nicotine [23]. This previous study also indicated that nicotine had an antibacterial effect on both smoking and non-smoking isolates at high concentrations (16–32 mg/ml). Furthermore, a recent study indicated that adding 1.0 mg/ml nicotine to *S. mutans* biofilm cultures increases the production of lactate by two folds compared to *S. mutans* biofilm cultures with zero nicotine [25,26]. The results of the present study demonstrated that there was a significant difference in biofilm formation between smoker and non-smoker isolates at almost all nicotine concentrations. There was a more significant increase in biofilm formation of smoker isolates at 1, 4, 8, 16 and 32 mg/ml compared to biofilm at the zero nicotine concentration. In this study, it was clear that *S. mutans* isolates from smokers are more influenced by high nicotine concentrations (up to 16 mg/ml) than non-smokers. In addition, this study indicated that planktonic cell growth was greater in non-smoking isolates at all nicotine concentrations compared to the planktonic cell growth of smoker isolates at the same nicotine concentrations. The possible mechanism of nicotine on enhancement of biofilm growth of *S. mutans* strains tested in the present study can be explained by a recent study that demonstrated the effect of nicotine on the expression of *Gbps* and *Gtfs* genes [22]. Interestingly enough, it was found that nicotine up-regulates the expression of *Gbps* and *Gtfs* genes of *S. mutans* planktonic cells and down-regulates *Gbps* and *Gtfs* of *S. mutans* biofilm cells [22]. Thus, an increase of planktonic cell attachment to biofilm results in increased growth of biofilm. In this study, there was not a significant relationship between the number of pack years smoked and biofilm formation of *S. mutans* isolates at all nicotine concentrations. The present study hypothesized that nicotine produces significant differences in biofilm formation between smoker and non-smoker *S. mutans* isolates. According to the study results, this hypothesis was confirmed. The rationale for this hypothesis was derived from preliminary data indicating that *S. mutans* can become tolerant to increased nicotine concentrations and this tolerance appears to be stable (unpublished data). This may allow smoker isolates to be able to respond more vigorously to higher nicotine concentrations than non-smoker isolates. This preliminary study suggested that *S. mutans* becomes adapted with stable resistance at high nicotine concentrations by some type of mutation and possible stable upregulation of antigen I/II after it had been passed at least three times on 0 mg/ml nicotine. The use of nicotine products increases the growth of *S. mutans* and may place tobacco users at risk for dental decay [27].

Results of this study suggest that there is more increased dental caries in smokers than non-smokers because of the significant increase of biofilm formation in the *S. mutans* smoker isolates compared to non-smoker *S. mutans* isolates. Further investigations in the effects of nicotine on different stages of biofilm formation of smoker *S. mutans* isolates can lead to understanding the complete picture and future development of more effective strategies and methods that prevent the development of dental biofilm and tooth decay in smokers. Also, to further learn the types of mechanisms and regulations that these strains use to tolerate high nicotine concentrations. The investigation of the effects of nicotine on smoker and non-smoker *S. mutans* isolates provides information that high nicotine concentrations can enhance more biofilm formation in smoker isolates than non-smoker isolates and this suggests a strong relationship between smoking and risk of developing dental decay.
